# The Role of Nano-domains in {1–011} Twinned Martensite in Metastable Titanium Alloys

**DOI:** 10.1038/s41598-018-30059-8

**Published:** 2018-08-09

**Authors:** Sangwon Lee, Chanhee Park, Jaekeun Hong, Jong-taek Yeom

**Affiliations:** Titanium Alloys Department, Metal Materials Division, Korea Institute of Materials Science (KIMS), Changwon, 51508 Republic of Korea

## Abstract

The formation mechanism of ${\{\bar{{\bf{1}}}{\bf{011}}\}}_{{\boldsymbol{\alpha }}{\boldsymbol{^{\prime} }}}$ type twinned α′-martensitic structures was investigated in titanium alloys, and in-depth characterizations of the microstructures were performed using scanning electron microscopy and transmission electron microscopy. The randomly distributed nano-domains nucleated by water quenching were sheared during the primary martensite transformation. Experimental results revealed that this sheared nano-domain interferes with the primary martensite transformation and induces a secondary martensite transformation. In terms of crystallography, the secondary martensite transformed from the sheared nano-domain has a ${\{\bar{{\bf{1}}}{\bf{011}}\}}_{{\boldsymbol{\alpha }}{\boldsymbol{^{\prime} }}}$ type twin relationship with the primary martensite. The growth of both martensites yielded a more twinned martensitic structure as the applied strain increased.

## Introduction

The martensitic transformation greatly influences the mechanical properties of titanium alloys. There are two martensitic transformation in Ti alloys: β → α′-martensite (hexagonal structure) and β → α″-martensite (orthorhombic structure). The α′-martensite transformation is observed in relatively lean alloy systems. There are two kinds of morphologies for α′-martensite: lath-type and plate-type martensite. The lath-type α′-martensite consists of bundles of parallel fine plate martensite. The boundaries of α′-martensite within the bundles are low angle dislocation boundaries^1^. As the solute content increases, transition of the α′-martensite morphologies occurs from bundles of α′-martensite to individual large plate α′-martensite (plate-type martensite)^2,3^. Some plate-type α′-martensites have ${\{\bar{1}011\}}_{{\rm{\alpha }}^{\prime} }$ type internal twins which originate from ${\{110\}}_{{\rm{\beta }}}$^3^. In highly alloyed systems, the structure of α′-martensite becomes distorted and orthorhombic α″-martensite is formed during quenching.

Previous studies reveal that (1) the impingement of secondary martensite to primary martensite and (2) the lattice invariant deformation generates ${\{\bar{1}011\}}_{{\rm{\alpha }}^{\prime} }$ type internal twins^1,4^. The formation of ${\{\bar{1}011\}}_{{\rm{\alpha }}^{\prime} }$ type internal twins is dependent on the composition. For example, the fraction of primary martensit that contain ${\{\bar{1}011\}}_{{\rm{\alpha }}^{\prime} }$ type internal twins increased as the Cr content increases to 5.5% in Ti-Cr binary system^4^. Even though the generation of internal twins is believed to accommodate the transformation strain^5^, the reason that internal twinning is observed only in relatively high alloy systems showing plate-type martensite is still unclear. The existing crystallography theory of the twinned martensitic transformation can not explain the dependence of composition^4^.

Ti-Nb-based Gum metal has been recently developed, which shows extraordinary mechanical properties including low elastic modulus, excellent cold ability, superelasticity and invar-like behaviour^6–9^. Tahara *et al*.^9^ reported that Ti-Nb exhibits a stress-induced martensite transformation while Ti-Nb-O suppress this transformation and promotes superelasticity behaviour. They noted that Ti-Nb-O contains nano-sized modulated structures which could affect the unique properties of Ti-Nb-based Gum metal, and suggested that these could originate due to the relaxation of oxygen local strain caused by $\{110\}{\langle 1\bar{1}0\rangle }_{{\rm{\beta }}}$ shuffling.

Martensite is related to the $\{11\bar{2}\}{\langle 111\rangle }_{{\rm{\beta }}}$ shear (lattice distortion) followed by $\{110\}{\langle 1\bar{1}0\rangle }_{{\rm{\beta }}}$ shuffle (lattice modulation). If only $\{110\}{\langle 1\bar{1}0\rangle }_{{\rm{\beta }}}$ shuffling occurs, the nanodomain (O′) with six shuffling modes can be generated^10^. O′ has orthorhombic symmetry (lattice parameters a = 0.317 nm, b = 0.448 nm and c = 0. 448 nm). The shear of $\{11\bar{2}\}{\langle 111\rangle }_{{\rm{\beta }}}$ in O′ can induce the martensite transformation, but only two martensite variants are possible for one shuffling mode. O′ is therefore known to suppress the martensitic transformation^9,10^. Zheng *et al*.^10,11^ investigated the effects of substitutional atom addition on the generation of O′. As the alloying content (Al, Zr, Sn) increased, $\{110\}{\langle 1\bar{1}0\rangle }_{{\rm{\beta }}}$ displacement becomes more favourable than $\{11\bar{2}\}{\langle 111\rangle }_{{\rm{\beta }}}$ displacement, resulting in O′ promotion even when the oxygen content is low.

Recently, Ti-4Al-4Fe-0.25Si-0.1O alloy has been developed for reduced production cost and enhanced mechanical properties compared to Ti-6Al-4V^12,13^. The alloy is in relatively high alloyed system compared to others showing the lath type martensite transformation like pure Ti^4^ and Ti-1Cu^1^. This alloy is metastable resulting in the α′-martensite transformation after quenching from 950 °C^12^. As the quenching temperature was decreased, the quenching-induced martensite transformation was inhibited because of an increase of β stability caused by a Fe partitioning from primary α to β^13^. Fe is strong β stabilizer element. In this condition, the strain or stress-induced α′-martensitic transformation occurred with the generation of ${\{\bar{1}011\}}_{{\rm{\alpha }}^{\prime} }$ type internal twins. Thus Ti-4Al-4Fe-0.25Si-0.1 O is a very suitable alloy for the study of plate type α′-martensite because it exhibit various α′-martensite transformation mechanism. In this study, we also found O′ in the β phase for water-quenched Ti-4Al-4Fe-0.25Si-0.1 O (wt.%). We noted that there is a special relationship between O′ and twinned martensitic structures. We carefully investigated the microstructure of Ti-4Al-4Fe-0.25Si-0.1 O by scanning electron microscopy (SEM) and transmission electron microscopy (TEM). Evidence that the internal twins originate due to O′ is introduced in this study.

## Results

**Figure 1:**
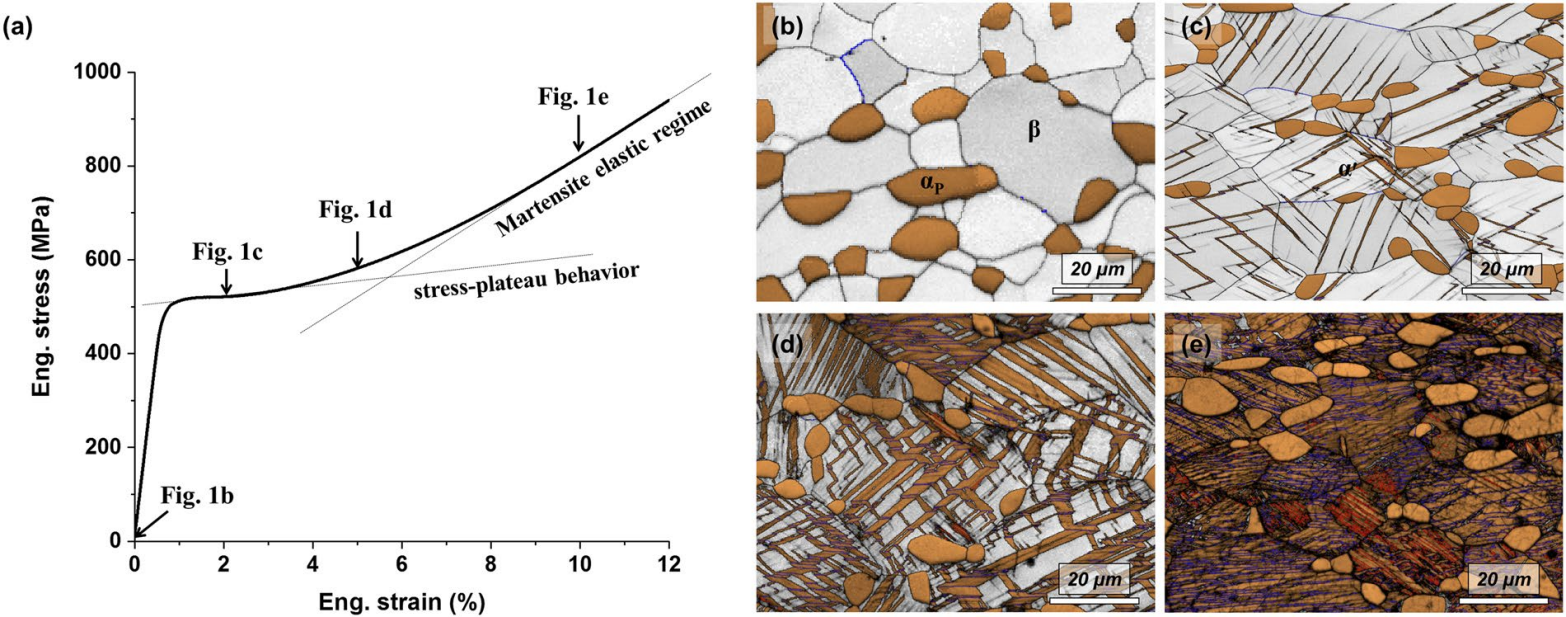
(**a**) Tensile behaviour of Ti-4Al-4Fe-0.25Si-0.1O alloy. Strain dependence of EBSD phase maps of Ti-4Al-4Fe-0.25Si-0.1O alloy strained to (**b**) 0%, (**c**) 2%, (**d**) 5% and (**e**) 10%. The blue and red boundaries of martensite are corresponded to twin and Burgers relationship, respectively.

Figure 1a shows the engineering tensile stress-strain curve of quenched Ti-4Al-4Fe-0.25Si-0.1 O. The alloy exhibits stress-plateau behaviour, which is frequently observed in Ti alloys showing stress-induced martensite transformations^14–17^. After stress-plateau behaviour occurs, an elastic deformation of transformed martensite generally occurs. Electron backscattering diffraction (EBSD) phase maps of Ti-4Al-4Fe-0.25Si-0.1 O strained to 0%, 2%, 5% and 10% are shown in Fig. 1b–e, respectively. The microstructure of the un-deformed sample consisted of primary α (α_p_) and β, and there was no trace of athermal martensite. As the applied strain increased, the stress-induced α′-martensite transformation occurred and its volume fraction increased. The Ti-4Al-4Fe-0.25Si-0.1 O strained to 10%, where it is in the elastic regime of martensite, showing that there is no retained β.

**Figure 2:**
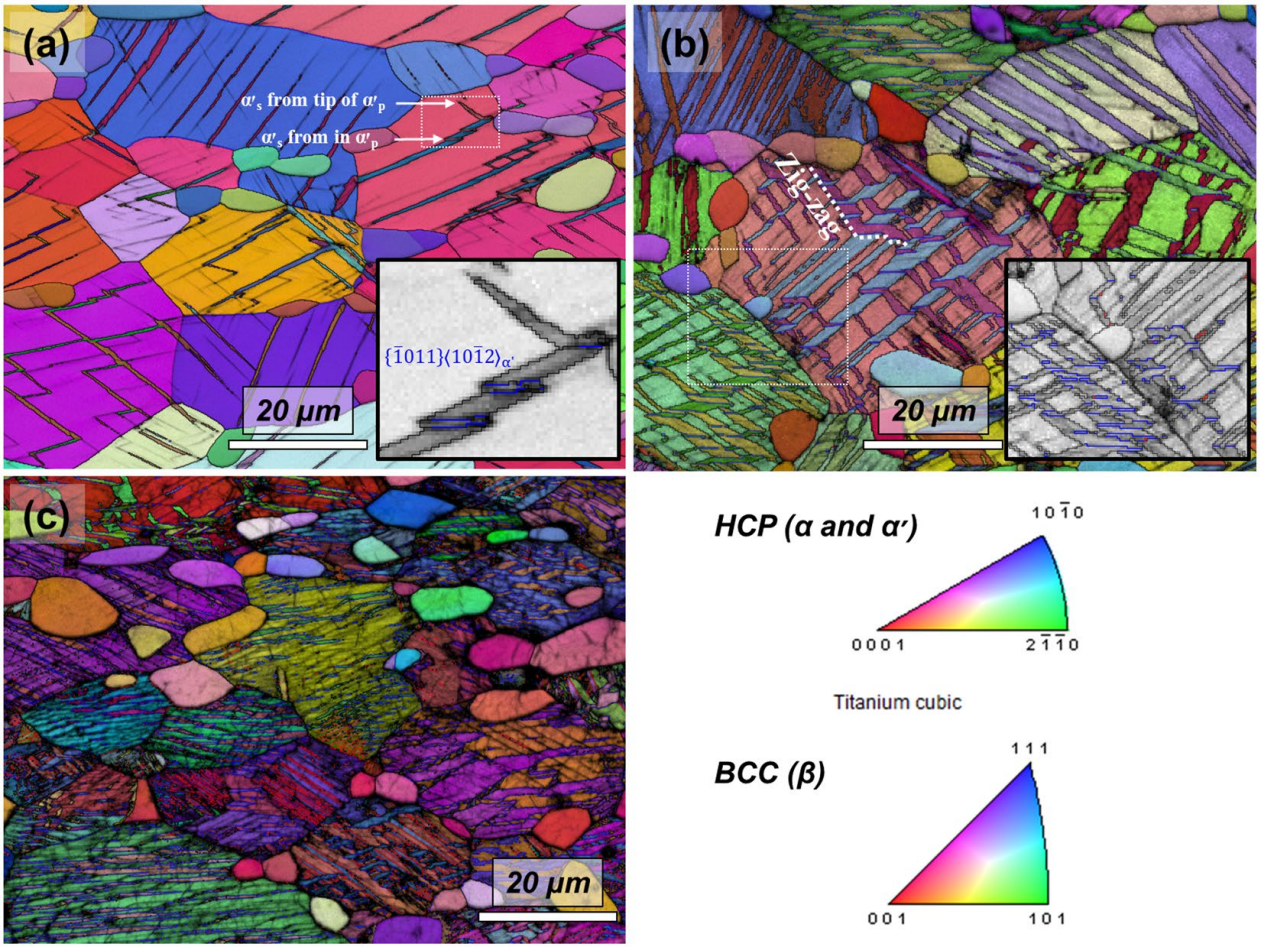
Strain dependence of EBSD inverse pole figure maps (parallel to the tensile direction) of Ti-4Al-4Fe-0.25Si-0.1O alloy strained to (**a**) 2%, (**b**) 5% and (**c**) 10%. The blue and red boundaries of martensite are corresponded to twin and Burgers relationship, respectively.

Figure 2a–c shows EBSD inverse pole figure maps corresponding to Fig. 1c–e. Normally, two variants of α′-martensite are observed in a β grain, the primary variant (α′_p_) which is directly transformed from β and a secondary variant (α′_s_) that is nucleated in α′_p_ or at the tip of α′_p_ as shown in Fig. 2a. The misorientation angle (blue line) between α′_p_ and α′_s_ is 57.22° which corresponds to a twin relationship, $\{\bar{1}011\}$ type internal twins^18^. As the applied strain increased, the growth of α′_s_ occurred and it crossed the other laths of α′_p_. The growth direction of α′_s_ is different between α′_p_ and β. This resulted in a zig-zag microstructure of martensite as shown in Fig. 2b. This zig-zag microstructure disappeared at a 10% strain, because almost all retained β was transformed to martensite at this point as shown in Fig. 2c. The microstructure after the strain reached 10% consisted of α_p_ and twinned α′-martensite (α′_p_ and α′_s_).

**Figure 3:**
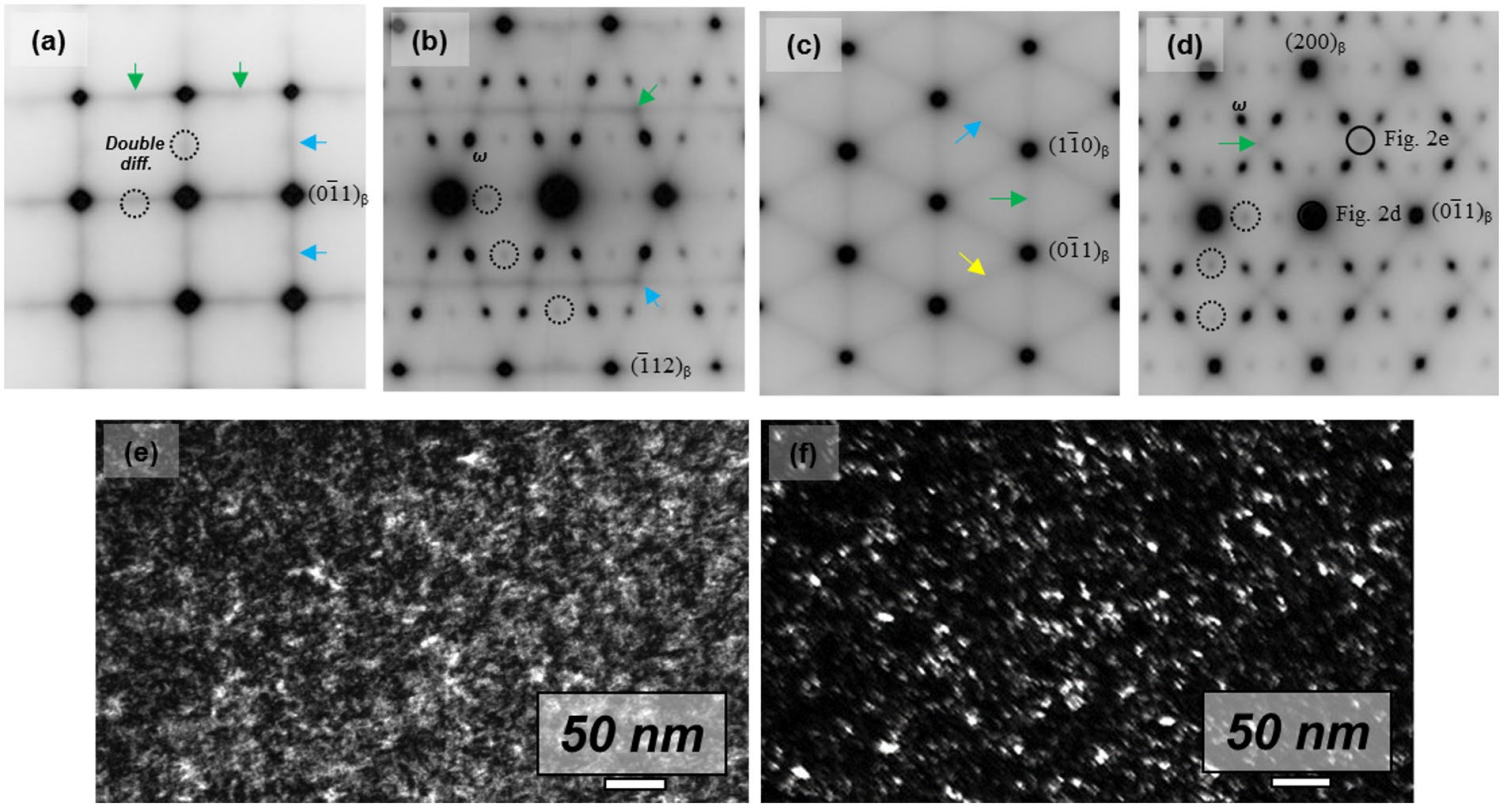
Selected area diffraction patterns of quenched Ti-4Al-4Fe-0.25Si alloy. The electron beam is parallel to (**a**) ${[100]}_{{\rm{\beta }}}$, (**b**) ${[311]}_{{\rm{\beta }}}$, (**c**) ${[111]}_{{\rm{\beta }}}$ and (**d**) ${[011]}_{{\rm{\beta }}}$. (**e**) Bright-field TEM micrograph of quenched Ti-4Al-4Fe-0.25Si-0.1O alloy. (**f**) Corresponding dark-field TEM micrograph showing the nano-domain.

TEM observations were carried out to investigate the origin of this twinned martensite. Figure 3a–d exhibits several selective area diffraction (SAD) patterns of a β grain of quenched Ti-4Al-4Fe-0.25Si-0.1 O. There are additional diffraction spots indicated by the arrows in each diffraction pattern of β. Even though some spots appear dim, bright and dark-field TEM micrographs clearly show that nano-sized particles (<10 nm) were nucleated in quenched Ti-4Al-4Fe-0.25Si-0.1 O. The spots of the nano-sized particles correspond to the nano-domain (O′), which is commonly reported in β-Ti species such as Ti-Nb based Gum metal^9,19^. Six possible variants of O′ are possible depending on the shuffling mode, as shown in Table 1. The variants of O′ are randomly distributed in annealed samples because all variants are energetically equivalent^19^. Figure 3a–d shows similar results in that several variants of O′ are observed. If an external stress (tensile or cold rolling) is applied to O′, preferential growth of an O′ variant occurs to release the applied stress^19,20^. This indicates that O′ is stress-dependent.

**Table 1:** All nano-domain variants depending on shuffling direction^17^.

Variants	Shuffling direction	Lattice correspondence		
		X	Y	Z
O′_1_	$(0\bar{1}1){[011]}_{\beta }$	${[100]}_{\beta }$	${[011]}_{\beta }$	${[0\bar{1}1]}_{\beta }$
O′_2_	$(011){[0\bar{1}1]}_{\beta }$	${[\bar{1}00]}_{\beta }$	${[0\bar{1}1]}_{\beta }$	${[011]}_{\beta }$
O′_3_	$(10\bar{1}){[101]}_{\beta }$	${[010]}_{\beta }$	${[101]}_{\beta }$	${[10\bar{1}]}_{\beta }$
O′_4_	$(101){[10\bar{1}]}_{\beta }$	${[0\bar{1}0]}_{\beta }$	${[10\bar{1}]}_{\beta }$	${[101]}_{\beta }$
O′_5_	$(\bar{1}10){[110]}_{\beta }$	${[001]}_{\beta }$	${[110]}_{\beta }$	${[\bar{1}10]}_{\beta }$
O′_6_	$(110){[\bar{1}10]}_{\beta }$	${[00\bar{1}]}_{\beta }$	${[\bar{1}10]}_{\beta }$	${[110]}_{\beta }$

**Figure 4:**
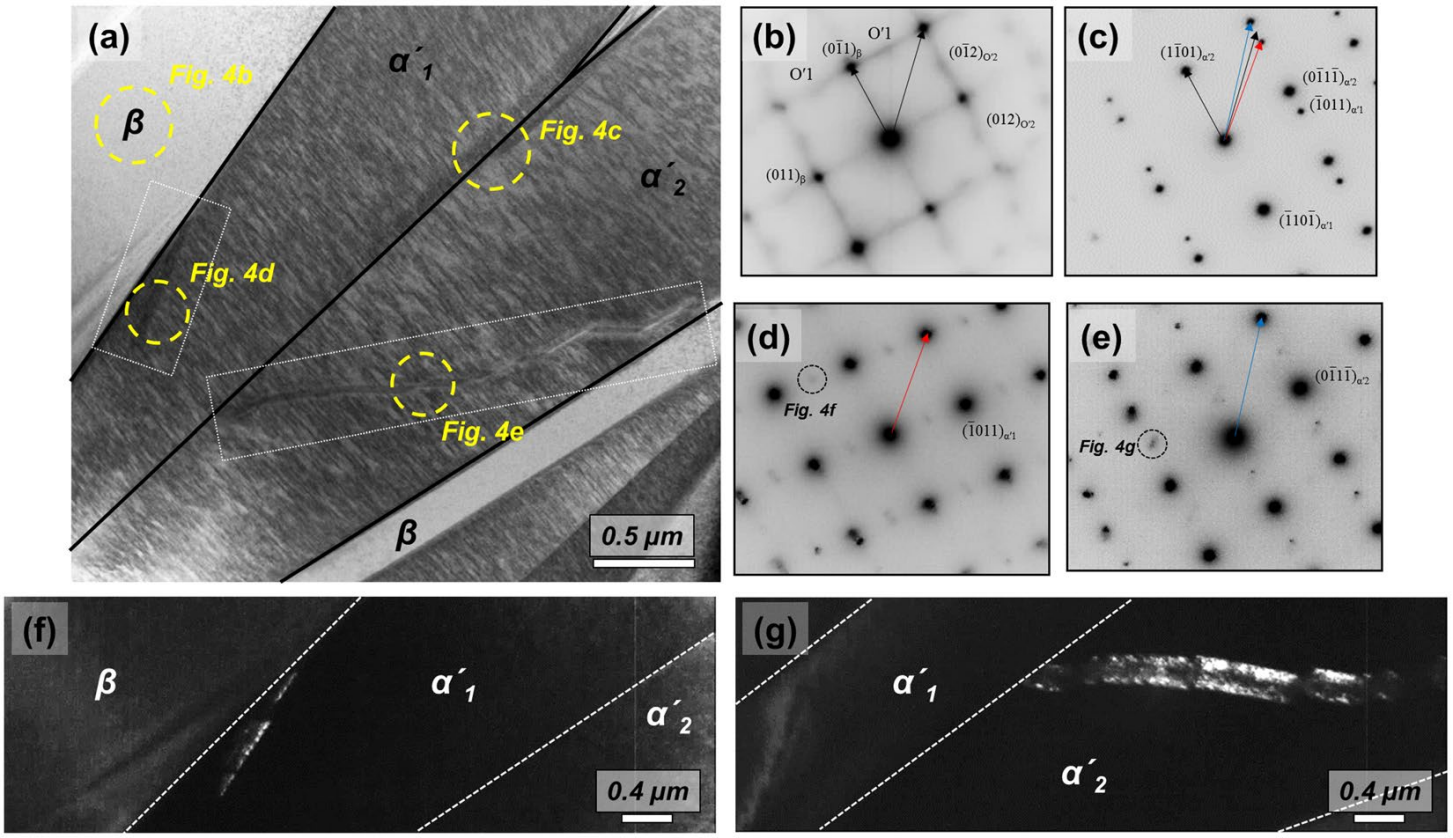
(**a**) STEM image of Ti-4Al-4Fe-0.25Si alloy stained to 2%. Selected area diffraction pattern of (**b**) β, (**c**) α′_1_ + α′_2_, (**d**) α′_1_ and (**e**) α′_2_ regime. The electron beam is parallel to ${[100]}_{{\rm{\beta }}}$. (**f**) Dark-field TEM micrograph showing an un-known phase in (**f**) α′_1_ and (**g**) α′_2_.

Figure 4a shows a bright-field STEM (scanning transmission electron microscope) image of Ti-4Al-4Fe-0.25Si-0.1 O strained to 2%. The electron beam was parallel to the ${[100]}_{{\rm{\beta }}}$ direction. The diffraction pattern of the β regime is shown in Fig. 4b. Two variants of O′ which are parallel to the ${\langle 100\rangle }_{{\rm{O}}^{\prime} }$ direction were observed. The O′ spots in the deformed sample are more clearly visible than in the un-deformed sample (Fig. 3a). Two variants of stress-induced α′_p_ (α′_1_ and α′_2_) in β were observed. The corresponding diffraction patterns of both α′_p_ as shown in Fig. 4c reveal that both α′_p_ have a ${\{\bar{1}011\}}_{{\rm{\alpha }}^{\prime} }$ type twin relationship. Based on Fig. 4c, the orientation relationships between α′_1,_ α′_2_ and β is

$$(0\bar{1}1){[100]}_{{\rm{\beta }}}//(1\bar{1}01){[01\bar{1}1]}_{{\rm{\alpha }}^{\prime} 1}//(1\bar{1}01){[\bar{1}011]}_{{\rm{\alpha }}^{\prime} 2}$$

Nishiyama *et al*.^21^ report that the ${\{\bar{1}011\}}_{{\rm{\alpha }}\text{'}}$ type twin plane comes from ${\{110\}}_{{\rm{\beta }}}$ assuming that the habit plane is ${\{334\}}_{{\rm{\beta }}}$ and Burgers orientation relationship hold good in the transformation. However, very few of these two α′_p_ in β were observed as shown in Figs 1 and 2. In addition, almost all misorientation angle between both α′_p_ (the grain boundaries between two α′_p_ are indicated by the red lines in Figs 1 and 2) were approximately 63.23° which corresponds to Burgers relationship^18^. The formation of twinned martensitic structures caused by these two α′_p_ should be minor. There are different contrasts in each α′_p_ as indicated by the white rectangle in Fig. 2a. Selective diffraction patterns of theses regimes shown in Fig. 4d,e, exhibiting additional diffraction spots as well as α′_p_. Dark-field TEM micrographs of additional spots clearly show that there are nano-sized lines (<400 nm) in α′_p_ as shown in Fig. 4f,g. In terms of shape, it is similar to the internal twins shown in the magnified section of Fig. 2a. In terms of diffraction patterns, the one variant of O′ observed in Fig. 4a appears to remain after the martensitic transformation. The crystal structure based on observed diffraction patterns are however neither α′_s_ nor O′.

## Discussion

**Figure 5:**
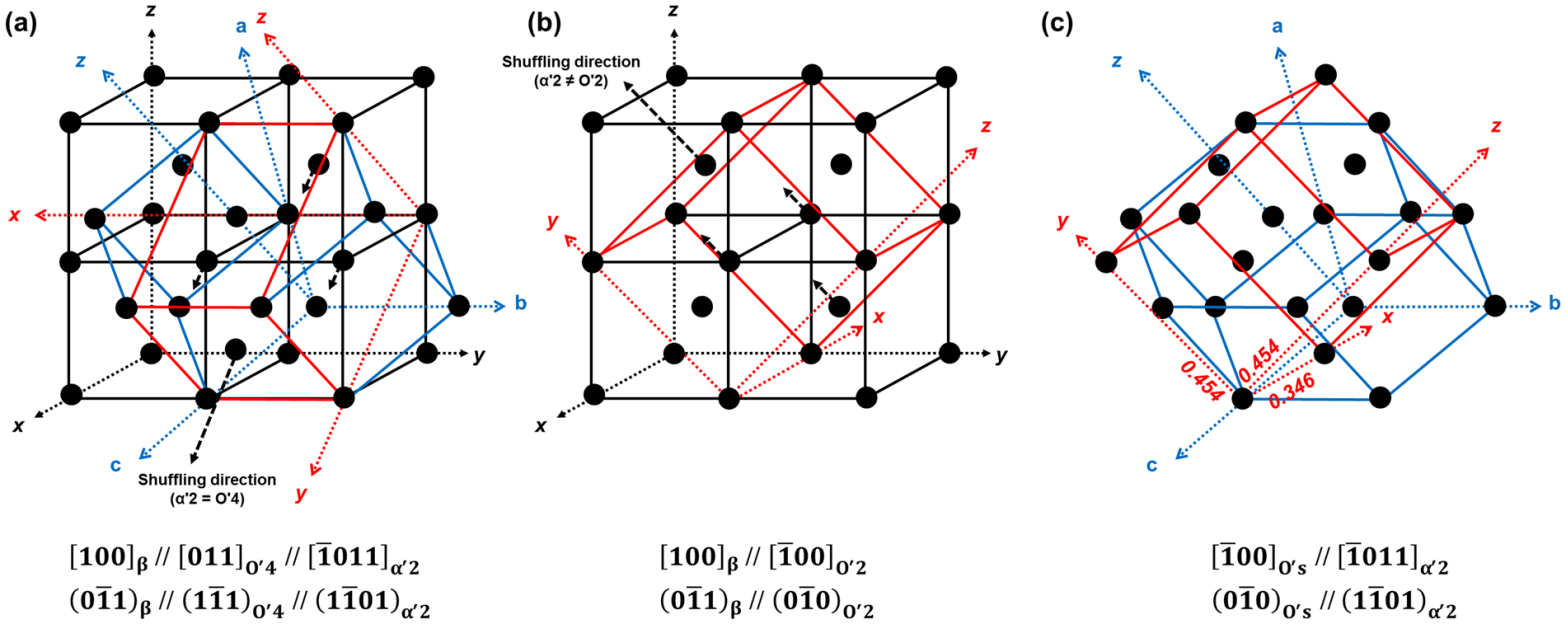
A schematic illustration exhibiting lattice correspondence: (**a**) β + O′_4_ + α′_2_, (**b**) β + O′_2_ and (**c**) α′_2_ + O′_s_.

$\{11\bar{2}\}{\langle 111\rangle }_{{\rm{\beta }}}$ shear (lattice distortion) is necessary to transform the martensite from O′. Each variant of O′ allows only two of the 12 possible variants of martensite^10^ because of its particular shuffling mode. This is one reason why O′ suppresses martensitic transformations. All variants of O′ are randomly distributed in annealed Ti alloys^19^. If the martensite transformation occurs in one of variants of O′, the other variants of O′ will be unfavourable for the transformation because they have different shuffling mode with the martensite. This unfavourable variant of O′ could remain in α′ in the sheared state. To investigate this, we drew an example of crystal structures of β, α′_2_ and O′ as shown in Fig. 5. The Burgers relationship between β and α′_2_ is present in Fig. 5a. The O′ variant which is favourable for the α′_2_ transformation is O′_4_ (Table 1). The diffraction spots of O′_4_ overlap with the β spots in the ${[100]}_{{\rm{\beta }}}$ zone axis of Fig. 1b. In the same manner, the O′ variant which is favourable for the α′_s_ transformation is O′_2_. The crystallography of β and O′_2_ is presented in Fig. 5b. During the α′_2_ transformation (=α′_p_ transformation), O′_2_ has an unfavourable orientation because the shuffling mode is different. Some O′_2_ could be sheared and become O′_s_ by $\{11\bar{2}\}{\langle 111\rangle }_{{\rm{\beta }}}$ type shearing for the α′_2_ transformation, as shown in Fig. 5c. The orientation relationships of Fig. 5 are as follows:

$$(0\bar{1}1){[100]}_{{\rm{\beta }}}//(1\bar{1}1){[011]}_{{\rm{O}}^{\prime} 4}//(1\bar{1}01){[\bar{1}011]}_{{\rm{\alpha }}^{\prime} 2}$$

$$(0\bar{1}1){[100]}_{{\rm{\beta }}}//(\bar{1}00){[0\bar{1}0]}_{{\rm{O}}^{\prime} 2}$$

$$(\bar{1}00){[0\bar{1}0]}_{{\rm{O}}^{\prime} {\rm{s}}}//(1\bar{1}01){[\bar{1}011]}_{{\rm{\alpha }}^{\prime} 2}$$

**Figure 6:**
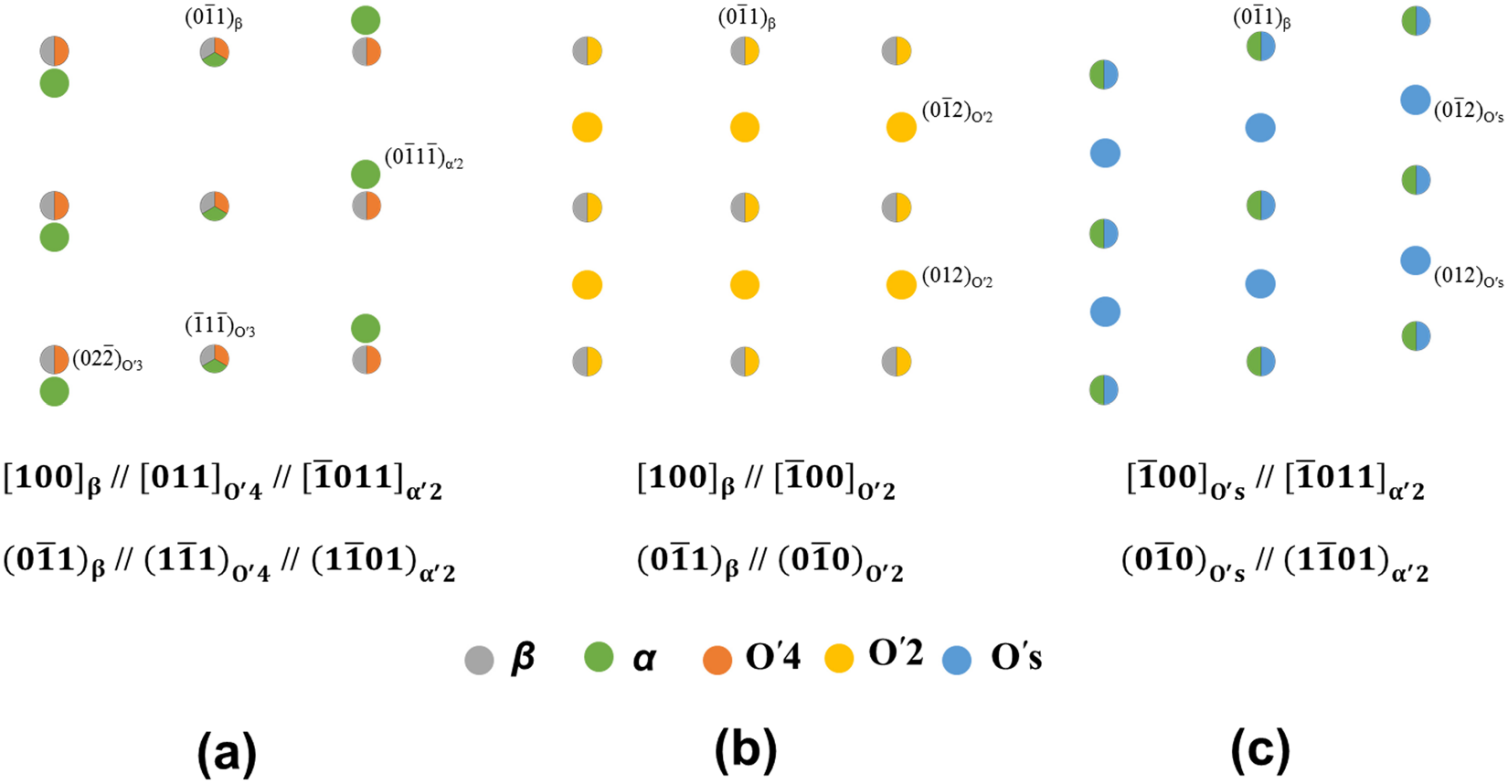
Simulated diffraction pattern in ${[100]}_{{\rm{\beta }}}$ zone axis showing (**a**) β + O′_4_ + α′_2_, (**b**) β + O′_2_ and (**c**) α′_2_ + O′_s_.

Figure 5a–c reveal that O′_s_ has a triclinic structure (angle α ≠ β ≠ γ). Based on the conventional lattice parameters of α (a = 0.294 nm and c = 0.468 nm), the crystal structure of O′_s_ was computed as (a = 0.346 nm, b = 0.454 nm, c = 0.454 nm, α = 80.71°, β = 86.28° and γ = 86.28°). Based on the orientation relationship and computed crystal structure of O′_s_, diffraction patterns were simulated as shown in Fig. 6. The simulated patterns of Fig. 6a–c are consistent with the experimentally measured patterns given in Fig. 4b–e.

**Figure 7:**
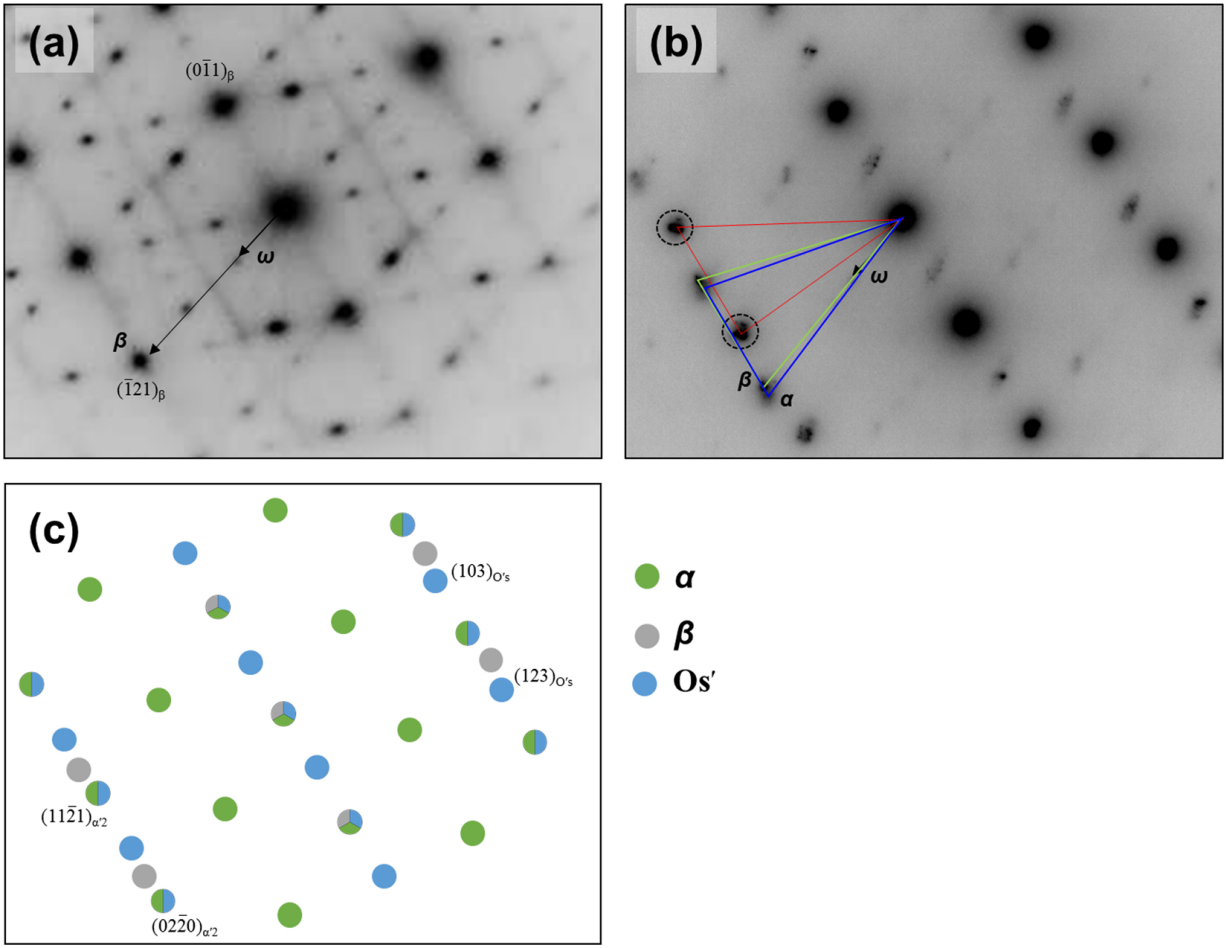
Selected area diffraction patterns of Ti-4Al-4Fe-0.25Si alloy strained to 2%: (**a**) β and (**b**) α′_2_ + O′_s_ regime. The electron beam is parallel to ${[311]}_{{\rm{\beta }}}$. Simulated diffraction pattern in ${[311]}_{{\rm{\beta }}}$ zone axis showing α′_2_ + O′_s_ + β.

**Figure 8:**
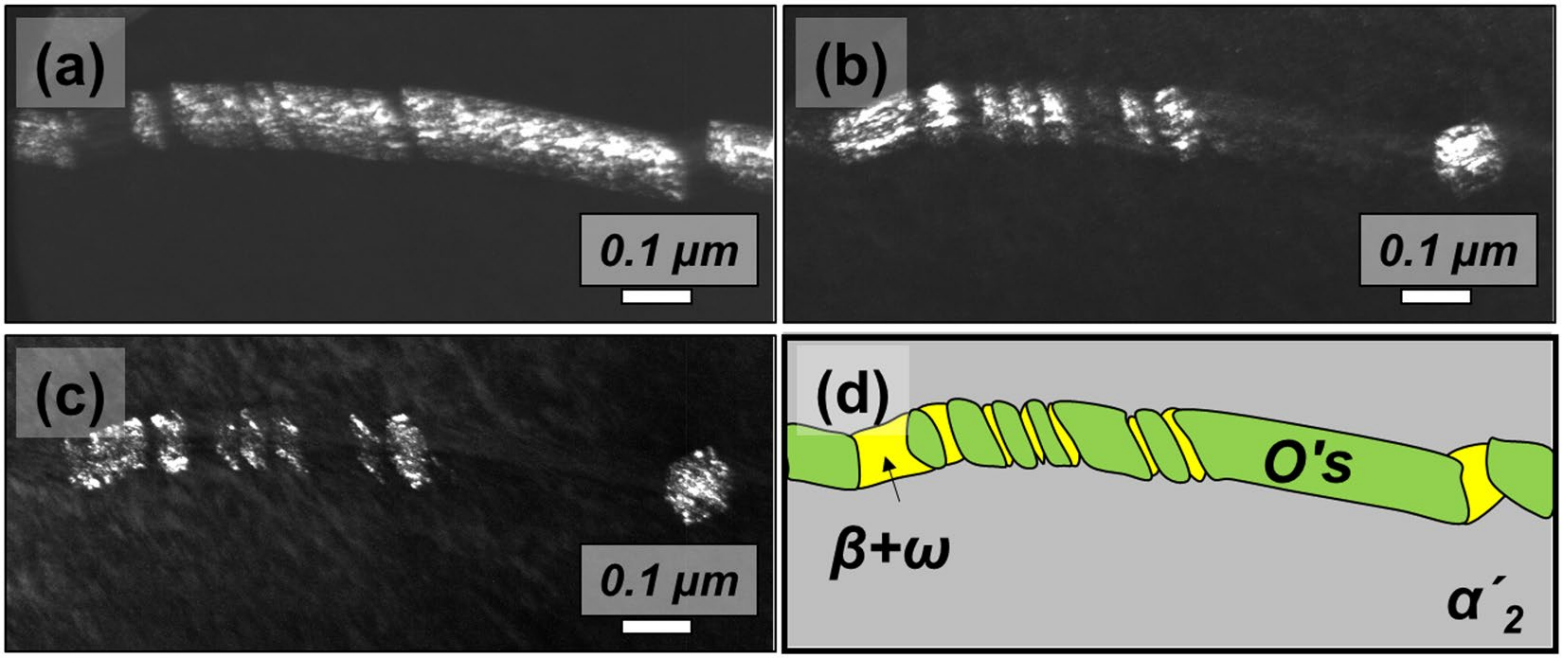
Dark-field TEM micrograph of Ti-4Al-4Fe-0.25Si alloy strained to 2% showing (**a**) O′_s_, (**b**) β and (**c**) ω. (**d**) Schematic to show the phase constituents in α′_p_.

The selective diffraction pattern of β matrix and α′_2_ + O′_s_ regime are shown in Fig. 7a–b respectively, where the specimen is tilted such that electron beam is parallel to the ${\langle 311\rangle }_{{\rm{\beta }}}$ direction of the matrix. Athermal ω was additionally observed in the β matrix. One thing to note is that diffraction spots of ω and β were also observed in the α′_2_ + O′_s_ regime. The simulated diffraction patterns of α′_2_, O′_s_ and β are similar to experimentally measured patterns. The deviation between Fig. 7b,c may be due to the fact that the zone axis of β in the α′_2_ + O′_s_ regime may be not parallel even though the zone axis of the β matrix is accurately parallel to the ${\langle 311\rangle }_{{\rm{\beta }}}$ direction. Dark-field TEM micrographs of O′_S_, β and ω (Fig. 8a–c respectively) clearly show that the β + ω regime is present between O′_s_. This un-transformed β + ω regime in α′_2_ reveals that different shuffling modes between α′_2_ (or O′_4_) and O′_s_ (or O′_2_) suppress the α′-transformation. If the β stability is high enough (as in Gum metal), the effect of O′ on the martensite transformation can be noticeable. To our knowledge, this is the first experimental evidence that O′ suppresses martensitic transformations.

**Figure 9:**
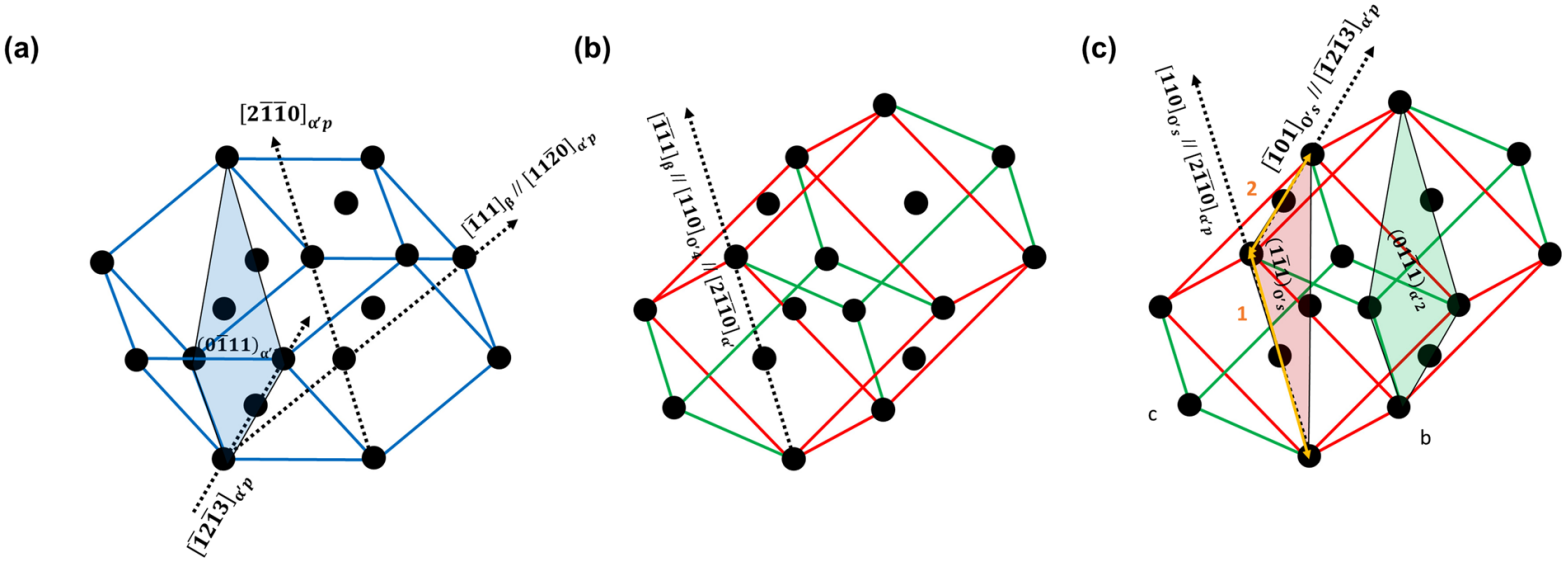
Schematic illustration exhibiting α′-martensite crystal structure transformed from (**a**) O′_4_, (**b**) O′_2_ and (**c**) O′_s_.

The crystallography of α′_p_ (or α′_2_) transformed from O′_4_ is presented in Fig. 9a. The α′_p_ had a Burgers relationship. The martensite transformed to O′_4_ could have a Burgers relationship in the same manner (Fig. 9b). Although α′_p_ martensite transformation from both O′ occur at the same time, the volume of twinned α′_p_ structure was small because the O′ variant is selected based on the applied stress. Tahara *et al*.^9^ and Kim *et al*.^19^ confirmed that variant selection of O′ occurs during cold rolling. Liu *et al*.^20^ showed that tensile loading induced variant selection of O′ while compressive loading suppressed O′ by means of an *in situ* high-energy X-ray diffractometer. Figure 4 showed no evidence of the variant selection of O′ because the performed pre-strain tests removed the residual stress. The variant-selected O′ is returned to the annealed state when the tensile loading is removed^20^. Therefore, the occurrence of variant selection of O′ during tensile loading could interfere with the nucleation of α′_p_ with other variants. The internal martensite (α′_s_) had a ${(\bar{1}011)}_{{\rm{\alpha }}\text{'}}$ type twin relationship with α′_p_. The sheared nano-sized domain (O′_s_) observed in α′_p_ may play a key role in the α′_s_ transformation and formation of the twinned martensitic structure. Figure 9c shows the crystallography of the possible transformation of martensite from O′_s_. ${[110]}_{{\rm{O}}^{\prime} {\rm{s}}}$ and ${[\bar{1}01]}_{{\rm{O}}^{\prime} {\rm{s}}}$ are parallel to ${[2\bar{1}\bar{1}0]}_{{\rm{\alpha }}^{\prime} {\rm{p}}}$ and ${[\bar{1}2\bar{1}3]}_{{\rm{\alpha }}^{\prime} {\rm{p}}}$, respectively. Thus, the ${(1\bar{1}1)}_{{\rm{O}}^{\prime} {\rm{s}}}$ is parallel to the ${(\bar{1}011)}_{{\rm{\alpha }}^{\prime} {\rm{p}}}$. To have a twin relationship, ${(1\bar{1}1)}_{{\rm{O}}^{\prime} {\rm{s}}}$ and ${(01\bar{1}1)}_{{\rm{\alpha }}^{\prime} {\rm{s}}}$ should be parallel each other. Even though the crystal structure of O′_s_ is not common in the literature, the lattice distance of ‘1’ and ‘2’ and angle between ‘1’ and ‘2’ in O′_s_ as shown in Fig. 9c is exactly the same as that of the martensite crystal structure. If the martensite transformation (α′_s_) occur from O′_s,_ α′_s_ can easily have a twin relationship with β through the invariant plane ${(1\bar{1}1)}_{{\rm{O}}^{\prime} {\rm{s}}}$ which minimizes the strain energy

**Figure 10:**
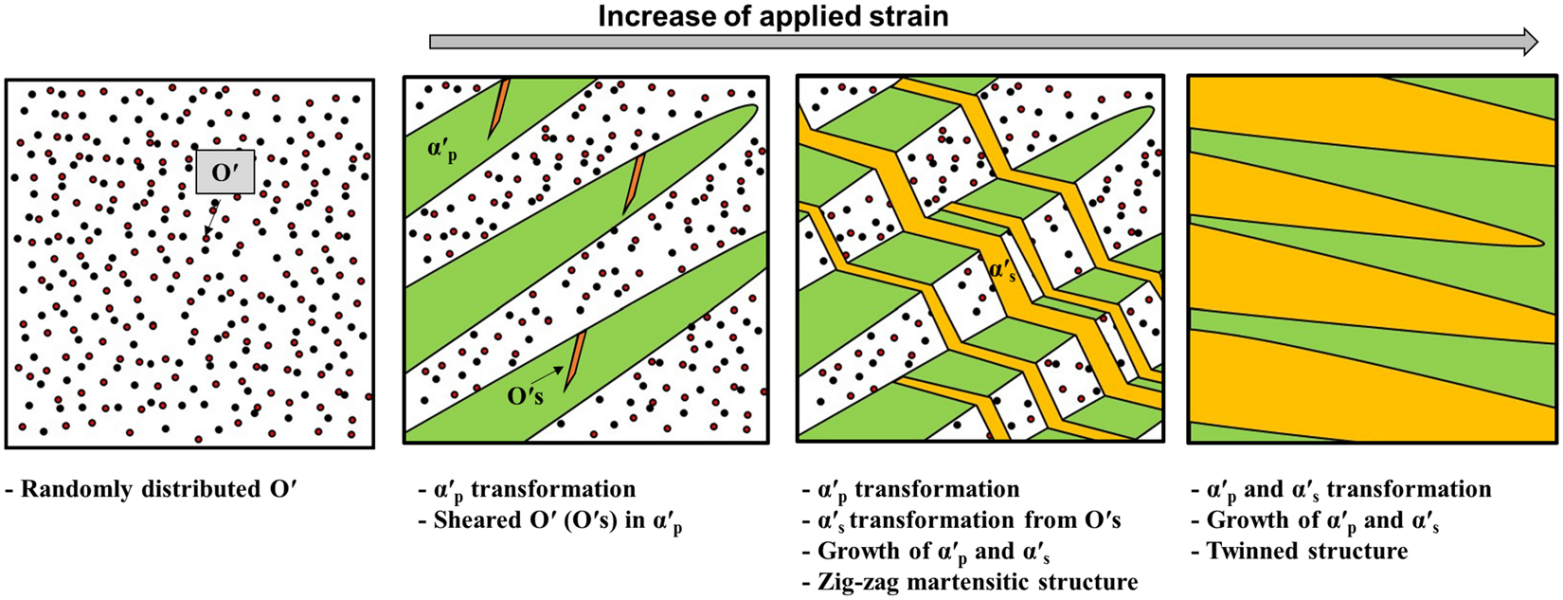
A schematic illustration exhibiting the procedure of forming the twinned martensitic structure.

Figure 10 illustrates the procedure of forming twinned martensite in metastable β alloys. All variants of nano-sized domains were randomly distributed during quenching. A variant of the nano-sized domain which is favourable to the applied stress induced a variant of the martensite (α′_p_) transformation. When the growth of α′_p_ pass through some nano-sized domains which have different shuffling directions with α′_p_, these domains are sheared (O′_s_) and still remain in α′_p_. As the applied strain increased, Oʹ_s_ is transformed to martensite (α′_s_). In terms of crystallography, α′_s_ has a twin relationship, $\{\bar{1}011\}10\bar{1}{2}_{{\rm{\alpha }}\text{'}}$with α′_p._ The growth of α′_s_ occurs and embeds in the retained β. The different growth directions of α′_s_ between α′_p_ and β result in an observed temporary zig-zag martensitic microstructure. As the applied strain increases further, β is perfectly transformed to two martensite (α′_s_ and α′_p_), and become a twinned martensitic structure.

Alloying elements change the possibility of $\{11\bar{2}\}{\langle 111\rangle }_{{\rm{\beta }}}$ shearing and $\{110\}{\langle 1\bar{1}0\rangle }_{{\rm{\beta }}}$ shuffling which are necessary for the martensitic transformation^14,22^. For example, Al and Zr addition in Ti-18Mo promoted $\{110\}{\langle 1\bar{1}0\rangle }_{{\rm{\beta }}}$ shuffling over the $\{11\bar{2}\}{\langle 111\rangle }_{{\rm{\beta }}}$, leading to the formation of nano-domains^22^. The variant selection of the nano-domain occurred depending on the stress state^9,19,20^. This selection determine the α′_p_^10^. The present study showed other variants of nano-domain which have different shuffling mode with the α′_p_ were sheared in martensite. This provided the nucleation site of twinned α′_s_. Namely, the generation of $\{\bar{1}011\}$-type internal twins in α′_p_ is dependent on the composition because the formation of nano-domains is dependent on the alloy composition. If the possibility of $\{11\bar{2}\}{\langle 111\rangle }_{{\rm{\beta }}}$ shearing is higher than that of $\{110\}{\langle 1\bar{1}0\rangle }_{{\rm{\beta }}}$ shuffling, the formation of nano-domains will be limited resulting in no $\{\bar{1}011\}$-type internal twins. If this transformation procedure (β → O′ → α′_p_) occur during quenching, various variants of athermal martensite in a prior β grain is expected to have each $\{\bar{1}011\}$-type internal twins.

In literature studies, nano-domains have been investigated in highly alloyed β titanium such as Nb-based Gum metal, Ti-23Nb-2Zr-0.7Ta-1.2 O (at.%)^19^, Ti-24Nb-4Zr-8Sn-0.1 O (wt.%)^20^ and Ti-26Nb-O (at.%)^23^. These studies reported that nano-domains affect the characteristic mechanical behaviour of Gum metals. The present work reveals that nano-domains were also observed in relatively lean alloyed and metastable titanium alloys showing an α′-martensite transformation. The nano-domain in metastable titanium alloy induced the twinned martensitic structure during deformation, and this twinned martensitic structure can greatly affect the mechanical properties of the alloy. For example, many Ti-based shape memory alloys have twinned martensitic structures in their annealed or low deformed states^24,25^. As the applied strain increases, detwinning and reorientation can occur; both of these influence the shape memory effect, superelasticity, and fatigue resistance of the alloy^26^.

In summary, the formation mechanism of twinned martensite caused by nano-domains was investigated by means of transmission electron microscopy and scanning electron microscopy and tensile tests in metastable Ti-4Al-4Fe-0.25Si alloy. The main conclusions are as follows:

1. Nano-domains were randomly distributed in the quenched alloys. During deformation, a nano-domain which has a variant favourable to the applied stress influenced the primary martensitic transformation.
2. Some nano-domains which had a variant unfavourable to the applied stress were sheared and remained in the primary martensite. Experimental results revealed that these domains interfered with the primary martensitic transformation and partially remained in the β in the primary martensite.
3. The sheared nano-domains acted as nucleation sites of the secondary martensite in primary martensite. The secondary martensite has a $\{\bar{1}011\}10\bar{1}{2}_{{\rm{\alpha }}\text{'}}$ twin relationship with the primary martensite.
4. As the applied stress increased, both primary and secondary martensite growth were promoted, and twinned martensitic structures were formed.

## Methods

The chemical composition of the titanium alloys used in the present work was Ti-4Al-4Fe-0.25Si-0.1 O (wt.%). Ingots were prepared by vacuum arc melting (VAR) followed by forging in the β or α + β regimes. The forged alloys were annealed to 880 °C for 6 h followed by water quenching. The β-transus temperature of the alloy was 925 °C^12,13^. The microstructure of the alloy were observed using a field-emission scanning electron microscope (FE-SEM; JEOL JSM-7100F) equipped with an Aztec EBSD system, and a field-emission transmission electron microscope (FE-TEM; JEOL JEM-2100F). The samples used for FE-SEM were mechanically polished with 400-grit sandpaper to remove surface defects and oxidation and then electropolished at a temperature of 0 °C in a solution containing 50 vol% CH_3_OH + 30 vol% C_4_H_9_OH + 20% HClO_4_ to remove any products of phase transformation resulting from the mechanical polishing. The sample used for FE-TEM were prepared by a focused ion beam (FIB; FEI Helios Nanolab 650) technique. ASTM E-8 sub-sized tensile specimens (gauge length: 10 mm and diameter: 2.5 m) were tested at 25 °C with a strain rate of 10^−3^ s^−1^ in an Instron universal tensile testing machine.

### Data availability

The datasets generated during and/or analysed during the current study are available from the corresponding author on reasonable request.

## References

[1] Williams JC, Taggart R, Polonis DH (1970). The Morphology and Substructure of Ti-Cu Martensite. Metall. Trans..

[2] Zangvil A, Yamamoto S, Murakami Y (1973). Electron microscopic determination of orientation. Metall. Trans..

[3] Nishiyama, Z. *Martensitic Transformation* (Academic Press, New York, 1978).

[4] Ericksen RH, Taggart R, Polonis DH (1969). The martensite transformation in Ti-Cr binary alloys. Acta. Metall..

[5] Guo S, Meng Q, Cheng X, Zhao X (2014). α′ martensite Ti-10Nb-2Mo-4Sn alloy with ultralow elastic modulus and high strength. Mater. Lett..

[6] Saito T (2003). Multifunctional Alloys Obtained via a Dislocation-Free Plastic Deformation Mechanism. Science.

[7] Shin S, Zhang C, Vecchio KS (2017). Phase stability dependence of deformation mode correlated mechanical properties and elastic properties in Ti-Nb gum metal. Mater. Sci. Eng. A.

[8] Plancher E, Tasan CC, Sandloebes S, Raabe D (2013). On dislocation involvement in Ti–Nb gum metal plasticity. Scr. Mater..

[9] Tahara M, Kim HY, Inamura T, Hosoda H, Miyazaki S (2011). Lattice modulation and superelasticity in oxygen-added β-Ti alloys. Acta Mater..

[10] Zheng Y (2016). The effect of alloy composition on instabilities in the β phase of titanium alloys. Scr. Mater..

[11] Zheng Y, Banerjee D, Fraser HL (2016). A nano-scale instability in the β phase of dilute Ti–Mo alloys. Scr. Mater..

[12] Lee SW (2017). Effects of TiFe Intermetallic Compounds on the Tensile Behavior of Ti-4Al-4Fe-0.25Si Alloy. Metall. Mater. Trans. A.

[13] Lee SW, Park CH, Hong JK, Yeom J-T (2017). Effect of solution treatment and aging conditions on tensile properties of Ti–Al–Fe–Si alloy. Mater. Sci. Eng. A.

[14] Kim H-S, Lim S-H, Yeo I-D, Kim W-Y (2007). Stress-induced martensitic transformation of metastable β-titanium alloy. Mater. Sci. Eng. A.

[15] Ahmed T, Rack HJ (1996). Martensitic transformations in Ti-(16–26 at%) Nb alloys. J. Mater. Sci..

[16] Li C, Wu X, Chen JH, van der Zwaag S (2011). Influence of α morphology and volume fraction on the stress-induced martensitic transformation in Ti–10V–2Fe–3Al. Mater. Sci. Eng. A.

[17] Ma X (2018). Strain rate effects on tensile deformation behaviors of Ti-10V-2Fe-3Al alloy undergoing stress-induced martensitic transformation. Mater. Sci. Eng. A.

[18] Lainé SJ, Knowles KM (2015). Deformation twinning in commercial purity titanium at room temperature. Philos. Mag..

[19] Kim HY, Wei L, Kobayashi S, Tahara M, Miyazaki S (2013). Nanodomain structure and its effect on abnormal thermal expansion behavior of a Ti–23Nb–2Zr–0.7Ta–1.2O alloy. Acta Mater..

[20] Liu J-P (2013). New intrinsic mechanism on gum-like superelasticity of multifunctional alloys. Scientific Rep..

[21] Nishiyama Z, Oka M, Nakagawa H (1966). {101̄1} Transformation Twins in Titanium. Trans. JIM..

[22] Zheng Y, Alam T, Banerjee R, Banerjee D, Fraser HL (2018). The influence of aluminium and oxygen additions on intrinsic structural instabilities in titanium-molybdenum alloys. Scr. Mater..

[23] Nii Y, Arima T, Kim HY, Miyazaki S (2010). Effect of randomness on ferroelastic transitions: Disorder-induced hysteresis loop rounding in Ti-Nb-O martensitic alloy. Phys. Rev. B.

[24] Ojha A, Sehitoglu H (2016). Critical Stresses for Twinning, Slip, and Transformation in Ti-Based Shape Memory Alloys. Shap. Mem. Superelasticity.

[25] Liu Y (2001). Detwinning Process and Its Anisotropy in Shape Memory Alloys. Proc. SPIE Smart Mater..

[26] Chai Y-W, Kim HY, Hosoda H, Miyazaki S (2009). Self-accommodation in Ti–Nb shape memory alloys. Acta Mater..

